# Inhibition of P2X7 Purinergic Receptor Ameliorates Cardiac Fibrosis by Suppressing NLRP3/IL-1*β* Pathway

**DOI:** 10.1155/2020/7956274

**Published:** 2020-05-21

**Authors:** Junteng Zhou, Geer Tian, Yue Quan, Junli Li, Xiaojiao Wang, Wenchao Wu, Miaoling Li, Xiaojing Liu

**Affiliations:** ^1^Department of Cardiology, West China Hospital, Sichuan University, Chengdu 610041, China; ^2^Laboratory of Cardiovascular Diseases, Regenerative Medicine Research Center, West China Hospital, Sichuan University, Chengdu 610041, China; ^3^Key Laboratory of Medical Electrophysiology of Ministry of Education, Institute of Cardiovascular Research, Southwest Medical University, Luzhou 646000, China

## Abstract

P2X7 purinergic receptor (P2X7R) has been implicated in several cardiovascular diseases. However, whether it regulates cardiac fibrosis remains elusive. Herein, its involvement in the development of cardiac fibrosis was examined using a transverse aortic constriction (TAC) mice model and cardiac fibroblasts (CFs) hyperstimulated by TGF-*β*1 for 48 hours. Results showed that TAC and TGF-*β*1 treatment increased the expression of P2X7R. Silencing of P2X7R expression with siP2X7R ameliorated TGF-*β*1 effects on fibroblasts activation. Similarly, P2X7R inhibition by Brilliant Blue G (BBG) reduced mRNA and protein levels of profibrosis markers, while the P2X7R agonist BzATP accelerated the TGF-*β*1-induced CFs activation. Moreover, it was found that TGF-*β*1-induced CFs activation was mediated by the NLRP3/IL-1*β* inflammasome pathway. BBG or siP2X7R treatment suppressed NLRP3/IL-1*β* pathway signaling. *In vivo*, BBG significantly alleviated TAC-induced cardiac fibrosis, cardiac dysfunction, and NLRP3/IL-1*β* activation. Collectively, our findings imply that suppressing P2X7R may limit cardiac fibrosis and abnormal activation of CFs.

## 1. Introduction

Myocardial remodeling is a hallmark feature of several cardiovascular diseases [1, 2]. Cardiac fibrosis is a pathologic condition caused by myocardial remodeling. The condition is characterized by disrupted cardiac morphology, extracellular matrix (ECM) deposition, and impaired cardiac function [3, 4]. Previous studies have shown that overstimulation of CFs triggers cardiac fibrosis. However, the molecular mechanisms of myocardial fibrosis caused by pressure overload have not been fully elucidated.

Evidence suggests that extracellular nucleotides, such as adenosine triphosphate (ATP), play a role in myocardial fibrosis [5, 6]. Under normal physiological conditions, a small amount of intracellular ATP is in the heart which stimulates erythrocytes, sympathetic nerves, endothelial cells, and skeletal and smooth muscle cells [7]. However, during hypoxia conditions, high concentration of glucose or abnormal shear stress triggers the release of a large amount of ATP into the interstitial space. Excessive extracellular ATP activates other purine signaling pathways leading to the generation of an amplification loop that promotes inflammation causing cellular injuries [6, 8].

P2X receptors are ligand-gated ion channels that respond to extracellular ATP. Seven P2X receptors (P2X1-P2X7) have been identified in the human heart [9]. Among them, P2X7R is the key mediator of inflammation [10]. In Crohn's disease or rheumatoid arthritis, this receptor triggers the production of cytokines associated with inflammation, interleukin-18 or interleukin-1*β* (IL-1*β*), and tumor necrosis factor-*α* (TNF-*α*) [11]. In the failing heart, ATP leakage from injured cells activates P2X7R on the cell membranes leading to the stimulation of NOD-like receptor pyrin domain-containing protein 3 (NLRP3). Subsequently, activated NLRP3 facilitates the assembly of a multiprotein complex named “the NLRP3 inflammasome,” consisting of NLRP3, an adaptor molecule termed apoptosis-associated speck-like protein containing a CARD domain (ASC) and caspase-1. Activated caspase-1 cleaves a 31 kDa precursor IL-1*β* (pro-IL-1*β*) into 17 kDa mature IL-1*β*. This causes severe inflammatory reactions aggravating cardiac dysfunction and fibrosis [12, 13].

Previous works have identified that P2X7R expression promotes myocardial infarction (MI), and inhibition of P2X7R alleviates elevated systolic blood pressure in rats with MI [14, 15]. Besides, P2X7R inhibitor conferred similar benefits in an experimental model of autoimmune myocarditis (EAM) [16]. However, changes in P2X7R expression and the impact of these changes on cardiac fibrosis induced by pressure overload are not known.

In this study, we postulated that P2X7R may promote cardiac fibrosis via the NLRP3/IL-1*β* pathway.

## 2. Materials and Methods

### 2.1. Animal Procedures, Transverse Aortic Constriction (TAC) Surgery, and Administration of an Antagonist of P2X7R (Brilliant Blue G)

All animal experiments were approved by the animal ethics committee of West China Hospital of Sichuan University (ethic number 2014003A). Male C57BL/6 mice (age, 6 weeks) were procured from the Experimental Animal Tech Co. of Weitonglihua (Beijing, China). The TAC procedure is widely performed to create models of cardiac fibrosis due to induced pressure overload [17]. The mice (at 8 weeks of age) were anesthetized with isoflurane (2.5% for induction, 1.0% for maintenance). The mice were then intubated using PE 90 tubing at the supine position on a heating pad for mechanical ventilation. The aortic arch was constricted with a 5-0 silk suture tied around a blunt 27-gauge blunt needle. The needle was removed after constriction. Sham-operated mice received similar surgical treatment with the omission of ligation. Three days after TAC or sham operation, the mice were further separated into two groups: the BBG group which received P2X7R antagonist BBG (i.p. 30 mg/kg body weight in 0.9% saline 10 ml/kg) (Sigma) three times per week and the saline group which received saline (i.p. 0.9% 10 ml/kg). Four weeks after surgery, echocardiography was performed on all mice to assess cardiac function. At the end of experiment, the mice were sacrifice, and the hearts were excised and then snap-frozen in liquid nitrogen at -80°C for further analysis.

### 2.2. Echocardiography

An echocardiography machine (iE33, Philips) fitted with a 35 MHz transducer was employed to evaluate cardiac remolding in mice four weeks following TAC operation. An experienced operator who was blinded to the treatment groups performed and analyzed echocardiography results. Briefly, the mice were exposed to isoflurane to anesthetize them and placed in a supine position on a heating pad. Images were captured in the short axis of the left ventricle to calculate internal wall dimensions during systole and diastole. From M-mode images, the thickness and dimensions of the left ventricle (LV) chamber were obtained. LV systolic function was determined by calculating ejection fraction (EF) and fractional shortening (FS).

### 2.3. Histological Analyses (Trichrome Staining, Sirius Red Staining, Scar Size Measurement, and Immunohistochemistry)

After echocardiographic evaluation, freshly isolated hearts were fixed with paraformaldehyde (4%), embedded in paraffin, and transversally sectioned (thickness, 4–5 *μ*m). For immunohistochemistry (IHC) staining, tissues were incubated with anti-periostin (Abcam, 1 : 200), and the staining was performed as per the methods described previously [18]. Masson's trichrome and Sirius red staining (Sigma) were performed following the manufacturer's instructions. Staining cross-section heart tissue images were recorded using a bright-field microscope (Leica). To evaluate the degree of cardiac fibrosis, the National Institutes of Health (NIH) ImageJ software was used to analyze images by comparing blue- or red-stained area (collagen) with the total area (10 sections were randomly chosen per heart, *n* = 6 hearts per group).

### 2.4. Cell Culture and Pharmaceutical Treatments

Neonatal rat cardiac fibroblasts were isolated from the hearts of decapitated Sprague-Dawley rats (age, 0-3 days) according to the methods described previously [19]. The CFs were grown in a culture flask with DMEM mixed with 10% FBS, and 100 U/ml of both streptomycin and penicillin. CFs at the second passaging were added into 6-well plates and cultivated to reach 70-80% confluence. The NRCFs were incubated for 4-6 hours with serum-free DMEM and then later treated with TGF-*β*1 (a known stimulator of NRCFs, 10 ng/ml, Sino Biological Inc.) for 48 h to induce fibroblasts activation and fibrosis. A subset of cells was treated with BBG (100 nM) after 2 h exposure to TGF-*β*1 or treated with BzATP (100 *μ*M) for 1 h at the end of stimulation.

### 2.5. Cell Transfection

Cells grown to 50-60% confluence were put in 6-well plates. They were transfected using transfection reagent riboFECT™ CP (RiboBio™, China). siRNAs targeting P2X7R (siP2X7R) were transfected into NRCFs for 24 hours and then treated with TGF-*β*1 for 48 hours. Individual siRNAs (100 nM, RiboBio™, China), riboFECT™ CP reagent and buffer, and DMEM were combined and then incubated for 15 minutes at room temperature. The product code numbers of each siRNA are shown in Table 1.

### 2.6. Immunofluorescence Methodology

Immunofluorescence staining on cells was performed as described previously [19]. The primary antibody for incubation was anti-*α*-SMA (Abcam, 1 : 200) or P2X7R (Alomone Labs, 1 : 200). Images were obtained using confocal microscopy and analyzed with the ImageJ software (NIH, USA).

### 2.7. Evaluation of Gene Expression

The real-time-PCR assay was performed to examine gene expression as per the methods described previously [18]. Briefly, total RNA was isolated from the mice hearts or cultured NRCFs using TRIzol (Invitrogen, USA). Next, the RNA was used as a template to synthesize cDNA with a reverse transcription (RT) kit (Toyobo, Japan). The qPCR was conducted using the SYBR Green Supermix kit (Bio-Rad, USA) on the BIO-RADCFX96™ Real-Time PCR Detection System and GAPDH served as the reference gene. The sequences of the primers used are displayed in Tables 2 and 3. The 2^−*ΔΔ*Ct^ threshold (Ct) method was used to calculate relative fold changes.

### 2.8. Western Blot Analysis

Mice tissues and cells were treated with (RIPA) lysis buffer to extract total proteins according to the protocol provided by the manufacturer. Subsequently, the BCA protein assay kit (Pierce, USA) was employed for protein quantification. An equivalent amount of cell or tissue lysates (25 *μ*g) were denatured and separated through SDS-PAGE (10%) and then transferred to 0.45 *μ*m PVDF membrane (Millipore, USA) using a Mini-PROTEAN III system (Bio-Rad, USA) [20]. Next, the membranes were blocked for 2 hours with 5% skimmed milk in TBST (tris-buffered saline) and Tween 20 solutions. After that, the membranes were incubated overnight (4°C) alongside the following primary antibodies: connective tissue growth factor (CTGF) (Abcam, 1 : 1000), NLRP3 (HuaBio, 1 : 1000), transforming growth factor *β* (TGF-*β*) (Abcam, 1 : 1000), periostin (Abcam, 1 : 1000), P2X7R (Affinity Biosciences, 1 : 1000), *α*-smooth muscle actin (*α*-SMA) (Abcam, 1 : 1000), IL-1*β* (HuaBio, 1 : 1000), caspase-1, and GAPDH (Cell Signaling Technology, 1 : 1000). The membranes were treated with horseradish peroxide-conjugated anti-rabbit or goat anti-mouse secondary antibodies (Zsgb Bio, 1 : 2000) for 2 hours at room temperature. The blots were visualized with enhanced chemiluminescence (ECL) substrate kit (Thermo, USA). Finally, the ImageJ software (NIH, USA) was used to evaluate the protein band intensities.

### 2.9. Assessment of Cell Proliferation with EdU Assay

The proliferative activity of cells was determined using 5-ethynyl-2-deoxyuridine (EdU) staining (Click-iT™ EdU Alexa Fluor™ 555 Imaging Kit, Thermo Fisher Scientific). Cellular organelles were stained by incubating cells with 50 *μ*M EdU for 1 hour, and the nuclei were counterstained with DAPI for 30 minutes. The percentage of EdU-positive cells was defined as the cell proliferation rate. And was calculated as the number of EdU-positive cells normalized to the number of DAPI-stained cells observed under a fluorescent microscope (Olympus). All assays were performed more than thrice.

### 2.10. Data Analysis

Data were analyzed using the SPSS 22.0 software and presented as mean ± SEM. For unpaired data, differences between groups were compared with Student's *t*-test or one-way analysis of variance (ANOVA). *P* value < 0.05 was taken as significant.

## 3. Results

### 3.1. Role of P2X7R in Pressure Overload-Induced Cardiac Fibrosis and CF Activation

**Figure 1:**
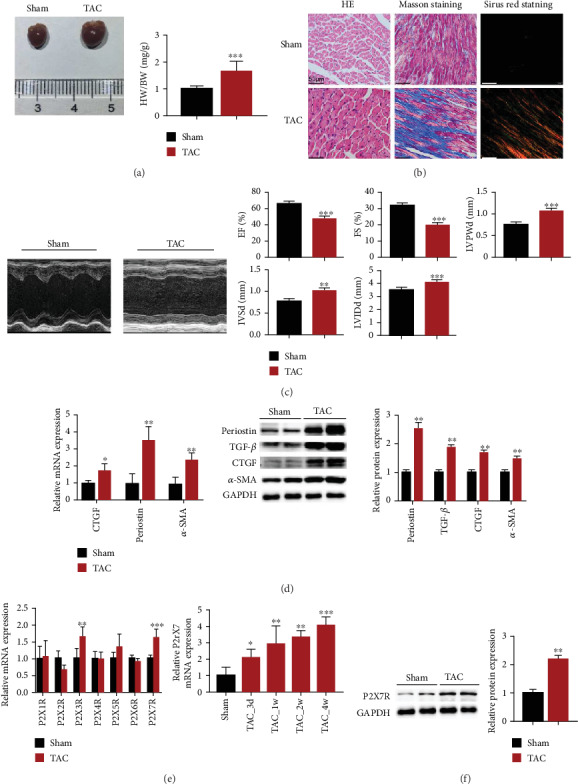
P2X7R was upregulated in the mice heart after TAC surgery. (a) Cardiac images and HW/BW (*n* = 8). (b) HE-, Sirius Red-, and Masson's trichrome-stained sections of the hearts following four weeks of TAC treatment (*n* = 6). (c) Representative images of an echocardiographic assessment of mice after TAC (4 weeks). Cardiac function indicators measured by echocardiography (LVEF (%), FS (%), LVPWd, IVSd, and LVIDd) in TAC- and sham-operated mice. (d) Expression of CTGF, periostin, and *α*-SMA mRNAs and proteins in heart cells (*n* = 6). (e) Gene expression of P2X receptors in TAC- and sham-operated mice (*n* = 6) at 4 weeks and gene expression of P2X7R in heart cells at 3 days, 1 week, 2 weeks, and 4 weeks after TAC- or sham-operated treatment (*n* = 6). (f) Expression of P2X7R protein in mouse heart cells (*n* = 6). Results are presented as means ± standard error of the mean. ^∗^ indicates *P* < 0.05, ^∗∗^ indicates *P* < 0.01, and ^∗∗∗^ indicates *P* < 0.001 versus sham.

Firstly, we explored the P2X receptor subtype(s) involved in TAC-induced cardiac fibrosis. Cardiac fibrosis following TAC was assessed using heart size, echocardiography indicators, HE, Masson's trichrome, and Sirius red staining. Figures 1(a)–1(c) show that TAC mice had higher heart weight relative to body weight (HW/BW) ratios, area of fibrosis and cross-sectional, left ventricular end-diastolic posterior wall thickness (LVPWd), LV internal diameter in diastole (LVIDd), and interventricular septal thickness (IVSd) but reduced fractional shortening (FS%) and ejection fraction (LVEF%) compared with the sham group. In addition, mRNA expressions of CTGF, periostin, and *α*-SMA were about 1.7, 3.5, and 2.3 times higher, respectively, in TAC mice relative to the sham group mice (Figure 1(d)). The periostin, TGF-*β*, *α*-SMA, and CTGF protein levels were also higher by 2.5-, 1.8-, 1.7-, and 1.5-fold, respectively, in TAC mice compared to the sham group mice (Figure 1(d)).

RT-PCR analysis was performed to detect expression of all P2X receptors mRNAs in the mouse hearts. Expressions of P2X3R and P2X7R mRNAs were higher in TAC relative to the sham mice hearts (Figure 1(e)). Among them, P2X7R began to increase three days after surgery, reaching the highest level in the 4th week (Figure 1(e)). Similarly, in the TAC group, the protein level of P2X7R was higher approximately by 2.1-fold in comparison to the sham group (Figure 1(f)).

**Figure 2:**
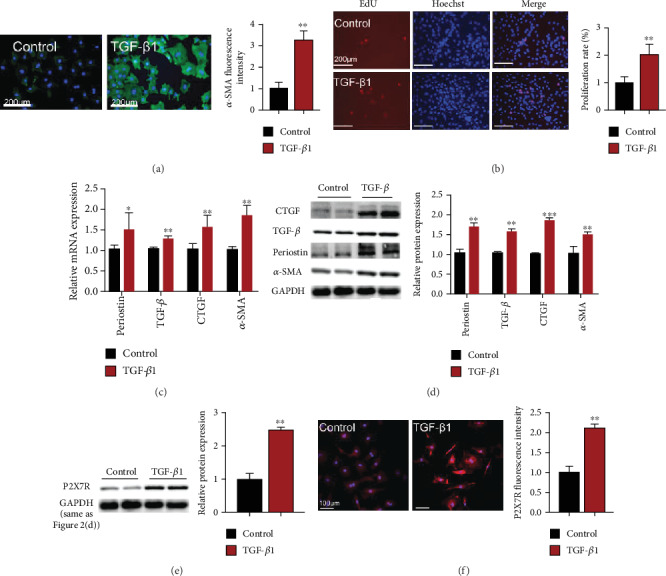
TGF-*β*1-treatment upregulated P2X7R in CFs. (a) Immunofluorescence images showing *α*-SMA expression in CFs (*α*-SMA (green) and nuclei DAPI (blue), *n* = 300). (b) Rate of cell proliferation (EdU-positive cells (red) and nuclei DAPI (blue), *n* = 120). (c) Expression of TGF-*β*, *α*-SMA, CTGF, and periostin mRNAs (*n* = 3). (d) Expression of CTGF, *α*-SMA, periostin, and TGF-*β* proteins (*n* = 3). (e) Western blot results showing P2X7R expression in CFs (*n* = 3). (f) Immunofluorescence images showing P2RX7 expression in CFs (P2X7R (red) and nuclei DAPI (blue), *n* = 60). Results are presented as means ± standard error of the mean; ^∗^ indicates *P* < 0.05, ^∗∗^ indicates *P* < 0.01, and ^∗∗∗^ indicates *P* < 0.001 versus control.

Subsequently, we examined the expression of P2X7R in neonatal rat cardiac fibroblasts following TGF-*β*1 (10 ng/ml for 48 h) treatment. The expressions of profibrosis markers' mRNAs, that is, periostin, CTGF, TGF-*β*, and *α*-SMA, were higher by 1.5, 1.3, 1.5, and 1.8 times, respectively, in CFs treated with TGF-*β*1 than in control group (Figure 2(c)). Also, the expressions of CTGF, TGF-*β*, periostin, and *α*-SMA proteins were higher by 1.7, 1.5, 1.8, and 1.5 times, respectively, in fibroblasts treated with TGF-*β*1 in comparison to the control group (Figure 2(d)). Moreover, *α*-SMA and P2X7R staining intensities were higher by 3-fold and 2.1-fold, respectively, in CFs treated with TGF-*β*1 than in control group (Figures 2(a) and 2(f)). The number of EdU-positive cells were 2-fold higher (Figure 2(b)) in NRCFs treated with TGF-*β*1 than control NRCFs. Meanwhile, the protein expression of P2X7R in fibrotic CFs was higher by almost 2.4 times relative to the untreated controls (Figure 2(e)). These findings imply that P2X7R expression elevated following pathological activation of NRCFs.

### 3.2. Suppression of P2X7Rs Protects CFs from TGF-*β*1-Induced Activation

**Figure 3:**
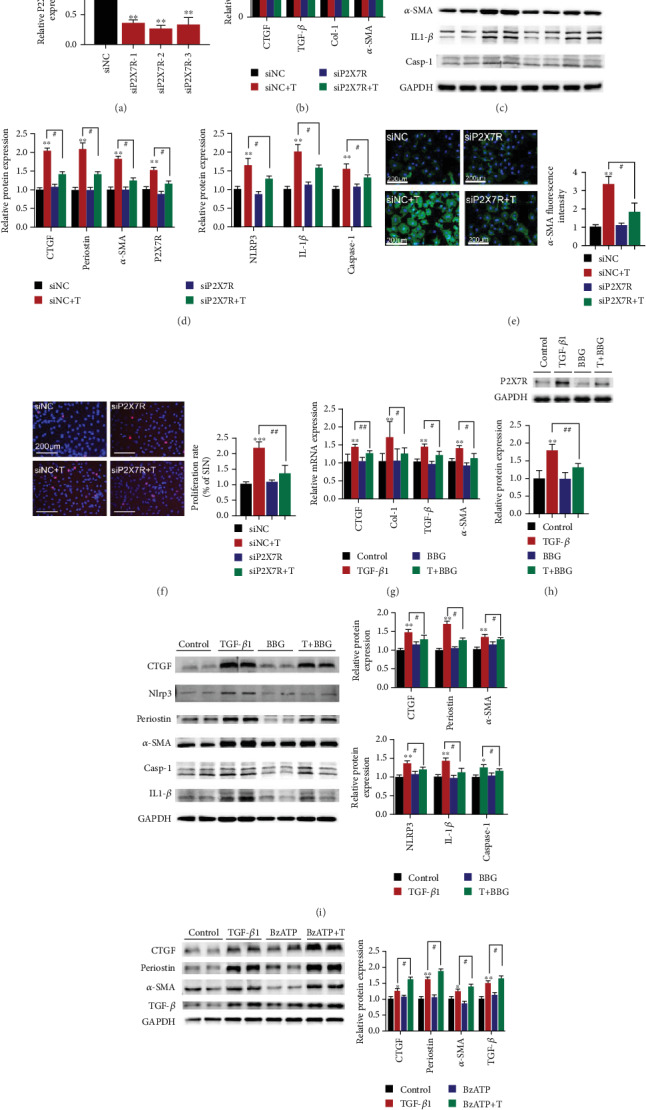
Knockdown of P2X7R alleviates the effects of TGF-*β*1 on CF activation. (a) Expression of P2X7R mRNA after siP2X7R transfection (*n* = 6). (b) Expression of collagen I, CTGF, *α*-SMA, and TGF-*β* mRNAs after transfection of CFs with siP2X7R (*n* = 6). (c, d) Expression of periostin, *α*-SMA, CTGF, P2X7R, NLRP3, IL-1*β*, and caspase-1 proteins after transfection of CFs with siP2X7R (*n* = 6). (e) Expression of *α*-SMA in CFs by immunofluorescence staining (*α*-SMA (green) and nuclei DAPI (blue), *n* = 300). (f) Changes in CF proliferation (EdU-positive cells (red) and nuclei DAPI (blue), *n* = 120). (g) Expression of collagen I, CTGF, *α*-SMA, and TGF-*β* mRNAs after treatment of CFs with BBG (*n* = 6). (h) Expression of P2X7R protein after BBG treatment (*n* = 6). (i) Expression of periostin, *α*-SMA, CTGF, NLRP3, IL-1*β*, and caspase-1 proteins after BBG treatment (*n* = 6). (j) Expression of periostin, *α*-SMA, TGF-*β* and CTGF proteins in activated CFs after BzATP treatment (*n* = 6). Results are presented as means ± standard error of the mean; ^∗^ indicates *P* < 0.05, ^∗∗^ indicates *P* < 0.01, and ^∗∗∗^ indicates *P* < 0.001 versus control or siNC; ^#^ indicates *P* < 0.05, ^##^ indicates *P* < 0.01, and ^###^ indicates *P* < 0.001.

To investigate whether P2X7Rs participated in TGF-*β*1-induced CF activation, siP2X7R silencing vector, and P2X7R inhibitor BBG were employed. Results showed that transfection with siP2X7R reduced mRNA expression levels of P2X7R by nearly 65% (Figure 3(a)), and that of CTGF (by 26%), TGF-*β* (by 28%), Col-1 (by 48%), and *α*-SMA (by 27%) compared with the control group cells (Figure 3(b)). At the protein level, CTGF, periostin, *α*-SMA, and P2X7R decreased by 27%, 28%, 31%, and 24%, respectively, relative to the control cells (Figure 3(c)). The relative fluorescence intensity of *α*-SMA in the CFs transfected with siP2X7R decreased by approximately 46% (Figure 3(e)). Moreover, the number EdU-positive cells in the group transfected with siP2X7R was fewer by nearly 38% compared to those of the control group (Figure 3(f)). The expression of P2X7R in cells treated with BBG after TGF-*β*1 stimulation was lower than that of cells treated with TGF-*β*1 alone (Figure 3(h)). As expected, treatment of cells with BBG after TGF-*β*1 stimulation reduced mRNA and protein levels of fibrosis markers (Figures 3(g) and 3(i)). In contrast, treatment with the P2X7R agonist, BzATP, increased the activation of CFs, as evidenced by 1.2-, 1.3-, 1.2-, and 1.2-fold elevation in periostin, CTGF, *α*-SMA, and TGF-*β* protein expression (Figure 3(j)). These data sets demonstrate that downregulating P2X7R ameliorates CF activation.

### 3.3. Downregulation of P2X7Rs Suppresses NLRP3/IL-1*β* Expression in TGF-*β*1-Induced CF Activation

Herein, we assessed the expression of three arms of NLRP3/IL-1*β* signaling pathways, including NLRP3, caspase-1, and IL-1*β* to elucidate whether NLRP3/IL-1*β* pathway was activated following TGF-*β*1 stimulation.  Figure 3(d) shows that these proteins were upregulated in the TGF-*β*1 group.

Next, we determined whether P2X7Rs regulated the NLRP3/IL-1*β* pathway. P2X7R knockdown in CFs treated with TGF-*β*1 led to lower levels of NLRP3 (20%), IL-1*β* (21%), and caspase-1 (15%) compared with their corresponding levels in the siNC+TGF-*β*1 group (Figure 3(d)).

Moreover, CFs cotreated with TGF-*β*1 and a P2X7R-specific inhibitor, and BBG had lower NLRP3, IL-1*β*, and caspase-1 levels in BBG+TGF-*β*1 than fibroblasts treated with TGF-*β*1 alone (Figure 3(i)).

### 3.4. Inhibition of P2X7Rs Suppresses Pressure Overload-Induced Cardiac Remodeling

**Figure 4:**
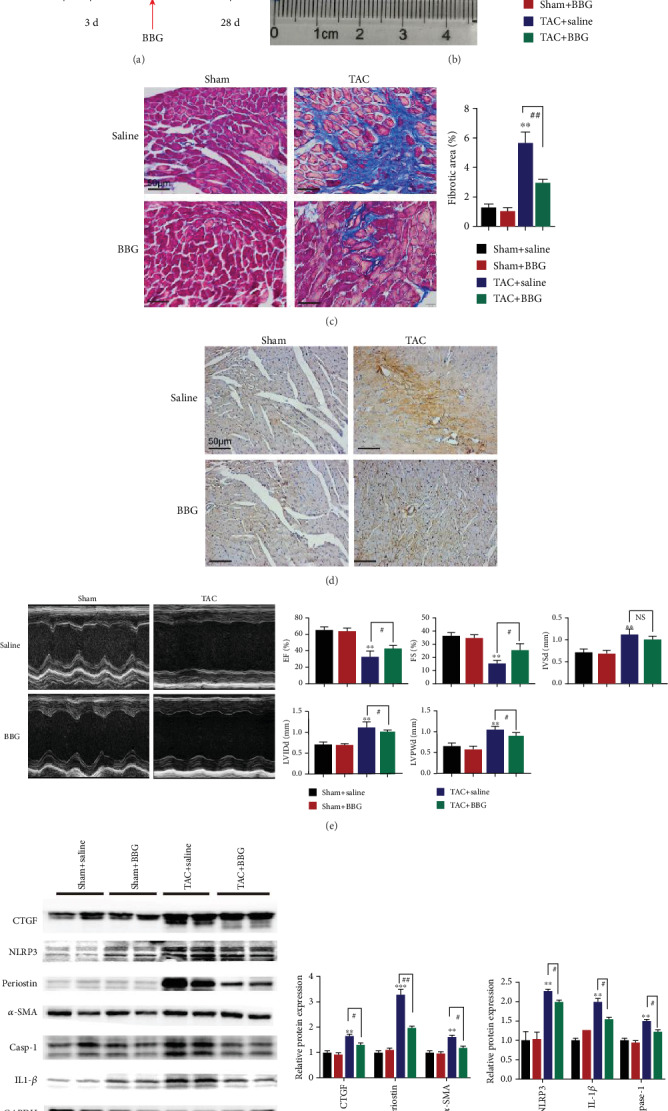
Inhibition of P2X7Rs suppresses cardiac fibrosis induced by pressure overload. (a–d) Effects of BBG on TAC-triggered fibrosis (a, b), histological (c, d), echocardiographic analyses (e). (a) Schematic diagram outlining the *in vivo* experiments. Mice were treated with BBG every two days at three days after TAC. (b) HW/BW and cardiac imaging results (*n* = 6). (c) Masson's trichrome staining (*n* = 6). (d) Periostin protein levels in ventricular tissues (periostin (brown) and nucleus (blue). (e) Representative echocardiographic images of mice after TAC (4 weeks); the cardiac function indicators evaluated by echocardiography (LVEF (%), FS (%), LVPWd, IVSd, and LVIDd) in TAC- and sham-operated mice. (f) Expression of periostin, *α*-SMA, CTGF, NLRP3, IL-1*β*, and caspase-1 proteins (*n* = 6). Results are presented as means ± standard error of the mean; ^∗^ indicates *P* < 0.05, ^∗∗^ indicates *P* < 0.01, and ^∗∗∗^ indicates *P* < 0.001 versus sham+saline. ^#^ indicates *P* < 0.05, ^##^ indicates *P* < 0.01, and ^###^ indicates *P* < 0.001.

Further experiments were performed to tests whether P2X7Rs were involved in pressure overload-induced cardiac fibrosis. Results showed that mice of the TAC+BBG group administered with BBG 2 days after TAC procedure showed a significantly lower HW/BW ratio, area of fibrosis, and number of periostin-positive cells than the mice in the TAC+saline group (Figures 4(a)–4(d)). In addition, treatment with BBG in the TAC group significantly improved EF (%), FS (%), LVIDd, and LVPWd compared with the TAC+saline treatment group (Figure 4(e)). Furthermore, the treatment with BBG decreased the protein expressions of CTGF, periostin, and *α*-SMA in the TAC group (Figure 4(f)).

We also examined whether pressure overload altered NLRP3/IL-1*β* signaling pathways in TAC mice. In the TAC+saline group, the NLRP3, IL-1*β*, and caspase-1 protein levels were higher by 2.2, 2.0, and 1.5 times, respectively, in comparison to the sham+saline group. In contrast, NLRP3, IL-1*β*, and caspase-1 levels were significantly lower by 11%, 25%, and 20%, respectively, in the TAC+BBG group relative to the TAC+saline group (Figure 4(f)).

These data suggest that P2X7R inhibition ameliorates cardiac dysfunction and cardiac fibrosis induced by pressure overload.

## 4. Discussion

**Figure 5:**
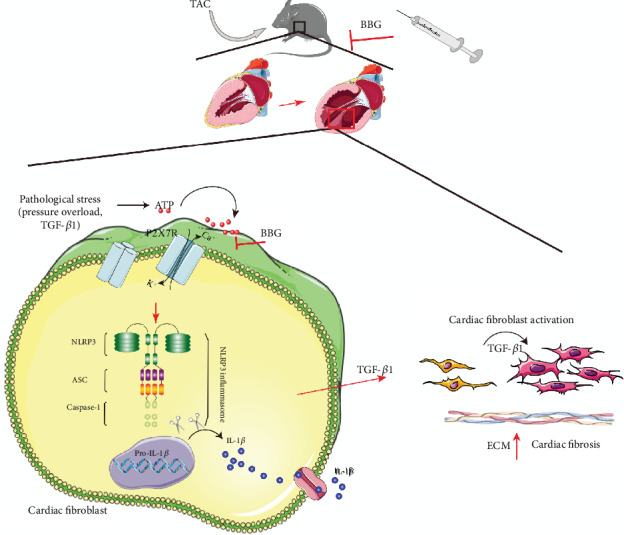
A schematic illustration of the mechanism of P2X7R in pressure overload-induced cardiac fibrosis and TGF-*β*1-induced CF activation. In TAC-induced injury, pathological stress, such as pressure overload and TGF-*β*1, triggers the release of ATP. Elevated ATP activates P2X7 receptors, contributing to P2X7-mediated NLRP3 inflammasome (NLRP3, ASC, and caspase-1) activation. This induces the release of IL-1*β*, causing severe inflammation that precipitates cardiac fibrosis and aggravates TGF-*β*1-induced CF activation. Moreover, inhibition of P2X7R with BBG inhibitor in TAC mice or CFs primed with TGF-*β*1 reduces fibrotic extracellular matrix (ECM) gene expression and cardiac fibrosis.

In this study, we examined whether P2X7R participates in cardiac fibrosis induced by pressure overload. The main results are shown in Figure 5.

Studies show that cellular activation, mechanical stimulation, or injuries induce ATP release into extracellular spaces, contributing to inflammatory reactions and fibrosis in injured heart cells [6, 21, 22]. Activation of the P2X receptor by nucleotides modulates cell migration, proliferation, inflammation, cytokines release, and necrosis [23]. P2X7R is an ATP ligand-gated cation channel that responds to extracellular ATP levels and plays a role in tissue fibrosis. In a silica-induced lung fibrosis mouse model, knockout of P2X7R in mice reduced lung fibrosis and inflammation. *In vitro* studies have shown that inhibition of the P2X7R prevented inflammatory responses in silica-treated fibroblasts and alveolar macrophages [24]. Tung et al. investigated the role of P2X7R in liver cirrhosis. They found that inhibition of P2X7R reduced hepatic inflammatory cytokines and suppressed TGF-*β* signaling pathway [25]. Similarly, P2X7R regulates heart responses to injury. Therefore, it plays a role in several heart conditions such as hypertension, acute myocardial infarction (AMI), and experimental autoimmune myocarditis (EAM) [14, 16, 26, 27]. However, the precise role of P2X7R in cardiac fibrosis is not known. Our results demonstrated that hearts from TAC mice exhibited elevated P2X7R expression. Although P2X7R is expressed in the human heart, it is clear whether it is expressed in cardiomyocytes and CFs [9]. Herein, the P2X7R protein was not detected in ventricular cardiomyocytes, but it was detected in endothelial cells and CFs [13, 28–30]. Herein, similar to *in vivo* findings, *in vitro* assays revealed that P2X7R was highly expressed in activated CFs.

The P2X7R antagonist, BBG, has been reported to exert therapeutic effects against lung and liver damage by preventing oxidative damage and inflammation. In a carrageenan- (CAR-) induced pleurisy mouse model, BBG decreased the assembly of the NRLP3/ASC/caspase-1 complex and iNOS, nitrotyrosine, and poly-ADP-ribosyl polymerase expression [31]. In a mouse model of acetaminophen- (APAP-) induced hepatotoxicity, a combination of celastrol and BBG prevented the hepatic antioxidant consumption (decreased superoxide dismutase and glutathione) and hepatocellular injury [32]. Elsewhere, administration of BBG decreased superoxide dismutase (SOD), malondialdehyde (MDA), TNF-*α*, and IL-1*β* concentration in pulmonary arterial hypertension (PAH) and ischemia-reperfusion- (IR-) induced lung models [33]. In our TAC mouse model, BBG affected several pathways controlled by P2X7R. We found that BBG partially attenuated the abnormal cardiac function caused by pressure overload. This outcome is consistent with that of P2X7R inhibitor, PPADS, both of which reduced cell death and cardiac fibrosis in mice with AMI [29]. Previous studies show that P2X7R was highly expressed in the cervical sympathetic ganglia in MI rats, but treatment with BBG or P2X7R siRNA restored P2X7R expression [34]. Consistently, we found that P2X7R mRNA and protein levels were elevated in CFs primed with TGF-*β*1. This effect was attenuated by P2X7R gene silencing. Similarly, BBG prevented CF activation by inhibiting P2X7R. However, P2X7R activation with BzATP agonist aggravated the effect of TGF-*β*1 on CFs. Thus, P2X7R participates in the pathogenesis of cardiac fibrosis and fibroblasts activation.

P2X7R is activated by high extracellular ATP [35]. Previous works suggest that ATP released from cardiomyocytes via pannexins initiates fibroblast proliferation and induces TGF-*β* expression in rat fibroblasts [36, 37]. We thus postulate that ATP released from cardiac cells accumulates in extracellular space through pannexins in mice subjected to TAC. This is evidenced by the upregulated expression of pannexin1 in TAC mice in this study (Figure S1). Studies have shown that TGF-*β*1 activation promotes cardiac fibrosis by inducing the transformation of fibroblasts into myofibroblasts leading to excessive collagen generation [38]. Other studies have shown that ATP released from cardiomyocytes in response to mechanical stretching stimulated purinergic receptors in CFs in a paracrine manner [39]. We speculate that stretch-triggered ATP release in the context of TAC may activate P2X7R receptors expressed on CFs, contributing to high collagen synthesis.

We also found that NLRP3/IL-1*β* signaling participates in P2X7R-mediated cardiac fibrosis. NLRP3/IL-1*β* is activated along with increased P2X7R expression. NLRP3 inflammasome regulates chronic sterile inflammation in response to intrinsic host signals released by injured cells. Activation of inflammasomes contributes to cleavage of pro-caspase-1 to its active form which further cleaves inactive pro-IL-1*β* into IL-1*β*. Activation of P2X7R is thus a vital step in the formation of NLRP3 inflammasome. In this way, P2X7R activation promotes cardiac dysfunction, cell death, and cardiac fibrosis [13, 40]. In a calcineurin-transgenic- (CNTg-) induced heart failure mice model, IL-1 receptor antagonist (IL-1-ra) inhibited cardiac inflammation and improved systolic function. Elsewhere, it was reported that the genetic ablation of Nlrp3 in CNTg mice reduced IL-1*β* and caspase-1 expression thereby improving cardiac function [12]. Surprisingly, mice with fibroblast-specific deletion of IL-1 receptor-1 showed improved cardiac function and lower expression of remodeling markers after MI compared with littermate controls. These results illustrate that inflammasome activation and IL-1*β* production contribute to cardiac dysfunction and abnormal morphology, especially in CFs [41]. In agreement with previous works, we showed that NLRP3 inflammasome was activated in response to TAC [42]. Most importantly, we showed that inhibition of P2X7R with BBG significantly ameliorated cardiac fibrosis and downregulated NLRP3, IL-1*β*, and caspase-1. A previous study indicated that the P2X7R antagonist ameliorated cardiac remodeling and prevented inflammasome formation following acute myocardial infarction (AMI) in mice [29]. Interestingly, other studies have shown that inflammasome formation occurs in CFs after cardiac injury which leads to the secretion of IL-1*β* [43]. Similarly, murine CFs exposed to lipopolysaccharide and ATP released IL-1*β*, indicating the involvement of P2X7R in CF activation [28]. In our study, the knockdown of P2X7R ameliorated CF activation and suppressed NLRP3/IL-1*β* pathway, as evidenced by reduced periostin, *α*-SMA, CTGF, NLRP3, and IL-1*β* levels. This is consistent with an earlier report in which P2X7R inhibitor or genetic deletion of P2X7R reduced the levels of IL-1*β* and NLRP3 in hepatic stellate cells treated with acetaldehyde, in cellular models of chronic alcoholic liver fibrosis [44]. Based on these observations, we speculate that the downregulation of P2X7R might inhibit the NLRP3/IL-1*β* pathway.

However, there are several limitations in this work. Although BBG is the most widely used P2X7R blocker, using P2X7R KO mice may more precisely demonstrate the function of P2X7R in cardiac fibrosis. Moreover, several lines of evidence have demonstrated that P2X7R expression is increased following activation of inflammatory cells such as macrophages, dendritic cells, mast cells, and T-lymphocytes [43, 45, 46]. Given that CFs are the principal producers of ECM, complex interactions between immune cells and CFs deserve further exploration.

We conclude that P2X7R activation promotes cardiac fibrosis. Also, we showed that inhibition of P2X7R may protect against CF activation and cardiac fibrosis by modulating the NLRP3/IL-1*β* pathway.

## Figures and Tables

**Table 1:** Sequences of siRNA to P2X7R.

Target	Sequences of SiRNA
siP2X7R-1	CCACAACUAUACUACGAGA
siP2X7R-2	GAGGAAUCAUGGGCAUUGA
siP2X7R-3	CGCUGUCAACCCAAAUACA
siRNA-NC	Provided by RiboBio™

**Table 2:** Rat RT-qPCR primer sequences.

Target mRNA	Sequence (5′⟶3′)
CTGF	Forward: CCCTGACCCAACTATGATGC
	Reverse: CCTTACTCCCTGGCTTTACG
GAPHD	Forward: AGTGCCAGCCTCGTCTCATA
	Reverse: GATGGTGATGGGTTTCCCGT
P2X7R	Forward: CGAAGTTAGTACACGGCATCTT
	Reverse: CTTGGCCTTCTGACTTGAGATAA
Periostin	Forward: ACGTCCTGGTGAAGTTGGTC
	Reverse: GGTGGATGACACCATTCTTC3
Col-1	Forward: ACGTCCTGGTGAAGTTGGTC
	Reverse: TCCAGCAATACCCTGAGGTC
TGF-*β*	Forward: TGAGTGGCTGTCTTTTGACG
	Reverse: ACTGAAGCGAAAGCCCTGTA
*α*-SMA	Forward: CCGAGATCTCACCGACTACC
	Reverse: ATGCCACAGGATTCCATACCC

**Table 3:** Mice RT-qPCR primer sequences.

Target mRNA	Sequence (5′⟶3′)
CTGF	Forward: GGACACCTAAAATCGCCAAGC
	Reverse: ACTTAGCCCTGTATGTCTTCACA
GAPHD	Forward: AATGGATTTGGACGCATTGGT
	Reverse: TTTGCACTGGTACGTGTTGAT
P2X7R	Forward: CAGCGGAAAGAGCCTGTTATC
	Reverse: TGGCCTTCTGACTTGACATAGTT
Periostin	Forward: TGGTATCAAGGTGCTATCTGCG
	Reverse: AATGCCCAGCGTGCCATAA
Col-1	Forward: TAAGGGTCCCCAATGGTGAGA
	Reverse: GGGTCCCTCGACTCCTACAT
TGF-*β*	Forward: CTTCAATACGTCAGACATTCGGG
	Reverse: GTAACGCCAGGAATTGTTGCTA
*α*-SMA	Forward: GGACGTACAACTGGTATTGTGC
	Reverse: TCGGCAGTAGTCACGAAGGA
Pannexin1	Forward: GCTGCACAAGTTCTTCCCCTA
	Reverse: CGCGGTTGTAGACTTTGTCAAG

## Data Availability

The accessibility data used to support the findings of this study were collected according with scientific research criteria and can be available from the corresponding author upon request.

## Supplementary Materials

Supplementary materials Figure S1: the mRNA level of pannexin1 in TAC- and sham-operated group.
