# Supplementary figures and images for: Inhibition of P2X7 Purinergic Receptor Ameliorates Cardiac Fibrosis by Suppressing NLRP3/IL-1β Pathway
Source: Oxid Med Cell Longev. 2020 May 21;2020:7956274. doi: 10.1155/2020/7956274 (PMC7261319; doi:10.1155/2020/7956274)

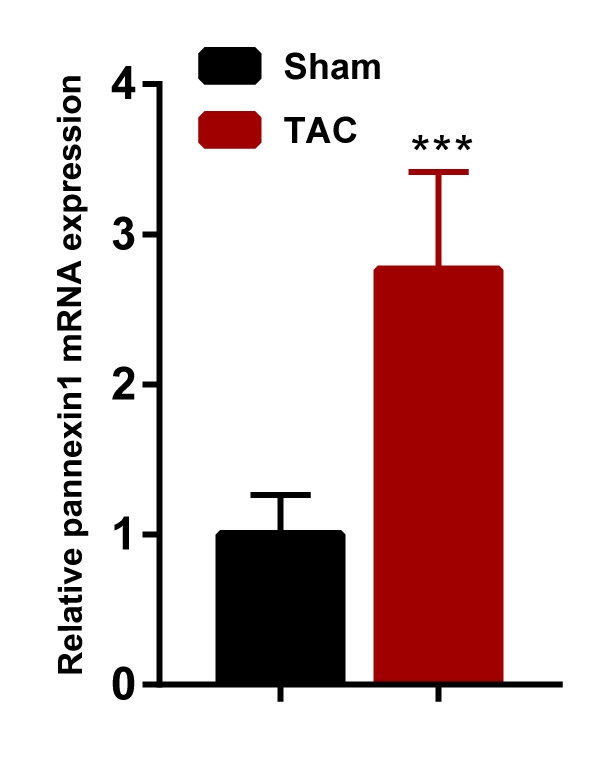

Supplement: Supplementary materials — Figure S1: the mRNA level of pannexin1 in TAC- and sham-operated group. [file 7956274.f1.tif]
